# Bullying and Cyberbullying in Minorities: Are They More Vulnerable than the Majority Group?

**DOI:** 10.3389/fpsyg.2016.01507

**Published:** 2016-10-18

**Authors:** Vicente J. Llorent, Rosario Ortega-Ruiz, Izabela Zych

**Affiliations:** ^1^Department of Education, University of CórdobaCórdoba, Spain; ^2^Department of Psychology, University of CórdobaCórdoba, Spain

**Keywords:** ethnic-cultural minorities, sexual minorities, bullying, cyberbullying, vulnerable groups, secondary education

## Abstract

Inclusion in education of all the children is necessary for the success, equality and peace among individuals and societies. In this context, special attention needs to be paid to the minorities. These groups might encounter additional difficulties which make them more vulnerable to be involved in bullying and cyberbullying. The current study was conducted with the objective of describing the involvement in bullying and cyberbullying of students from the majority group and also from sexual and ethnic-cultural minorities. The second objective was to explore if the implication is predicted by the interaction with gender, grade and the size of the population where the schools are located. It is an *ex post facto* transversal descriptive study with a survey on a representative sample of adolescents enrolled in the Compulsory Secondary Education in the south of Spain (Andalusia). The survey was answered by 2139 adolescents (50.9% girls) in 22 schools. These participants were selected through the random multistage cluster sampling with the confidence level of 95% and a sampling error of 2.1%. The results show that the minority groups, especially sexual minorities, are more involved in bullying and cyberbullying. Regression analyses show that being in the majority or a minority group predicts a small but significant percentage of variance of being involved in bullying and cyberbullying. Results are discussed taking into account the social vulnerability of being a part of a minority group and the need of designing educational programs which would prevent this vulnerability thorough the inclusion in education. There is a need for an educational policy that focuses on *convivencia* and *ciberconvivencia* which would promote the social and educational development of all the students.

## Introduction

The economic growth and richness in the developed world are apparently increasing in the last years. At the same time, the gap between the rich and the poor seems to increase and many groups are becoming even more vulnerable to violence and social exclusion (UNESCO, 2015a). Thus, the World Education Forum (UNESCO, 2015b) highlights the importance of the inclusive education which would lead to academic success, equity and peace for individuals and societies. Many research studies and contributions in educational settings try to optimize the educational system and achieve school success for all the students including the most vulnerable groups (Ainscow, 2012). Researchers point out that minorities might have some difficulties in accessing and following formal education and that these difficulties should be solved making it possible that all the students have equal opportunities to be included in the system (Booth and Ainscow, 2002). This led to the development of the inclusive education perspective (Ainscow et al., 2006) according to which schools should guarantee that no student is left behind because of marginalization, exclusion or failure.

Different minorities are present in the modern societies and therefore also in schools. Among them, new research lines focus on diverse ethnic-cultural groups and sexual minorities. For example, in some European countries, there are permanent ethnic-cultural minorities such as Gypsies and new minorities of first and second generations of immigrants (Llorent-Bedmar, 2013). This diversity is increased by the migrations of the refugees proceeding from the zones in ongoing armed conflicts (Sanahuja, 2014). Although sexual minorities have always been present in the school settings, the first studies on the topic were conducted at the end of the XX century (Kosciw et al., 2014). It has been pointed out that the inclusion of these groups is still insufficient and that there are still cases of discrimination in schools (Graybill and Proctor, 2016).

Besides acquiring academic knowledge, modern schools are places in which students are intended to be educated to gain skills for life. In this context, interpersonal relationships among the members of the school communities are a key factor for the education of future citizens of the world. A Spanish word *convivencia*^1^ is a term that describes relationships that are positive and based on moral principles of solidarity and respect, where rules are established and followed and conflicts are solved through democratic dialogue (Ortega-Ruiz and Zych, 2016). Given the fact that interpersonal relationships among young people are also initiated and maintained through the new technologies, a new line of studies on *convivencia* in the cyberspace (*ciberconvivencia*) has been recently started (Ortega-Ruiz et al., 2014). There are many authors who suggest that positive school-climate and good interpersonal relationships are also possible in multicultural and diverse school settings (Byrd, 2015). Thus, successful education should include *convivencia* and *ciberconvivencia* among different minority and majority groups.

*Convivencia* and *ciberconvivencia* are not always present in the schools. In some cases, negative relationships among different members of the school community can evolve in aggressive behaviors and violence (Ortega-Ruiz, 2015). School bullying is an extremely damaging type of violence present in schools. It is a long-term intentional aggressive behavior perpetrated by some students on their peers who cannot defend themselves (Smith and Brain, 2000). It is perpetrated under a dominance-submission scheme and imbalance of power (Ortega, 2010). Cyberbullying has been defined as bullying perpetrated through the electronic devices (Tokunaga, 2010), it is also intentional, frequent and long term and the victim has difficulties in defending him or herself from this kind of violence (Smith et al., 2008). Both phenomena were found to be correlated and there is overlap between the two (Del Rey et al., 2012; Baldry et al., 2016).

Cross-cultural research shows that bullying and cyberbullying are present in different countries (Smith et al., 2002; Craig et al., 2009; Ortega et al., 2012; Baldry et al., 2015). Many studies were conducted to compare their prevalence among genders, age groups and some focused also on the school location such as rural or urban settings or population size. Given the fact that research on bullying is conducted in schools, age and grade are often used interchangeably. A systematic review of theoretical studies on bullying and cyberbullying (Zych et al., 2015a) shows that the results are inconclusive and that there is no specific profile of involvement in this kind of violence. Meta-analytic results including 153 empirical studies on bullying (Cook et al., 2010) showed that boys were more involved in all the bullying roles (perpetration, victimization and bully/victim), although with small gender difference. This study also found weak positive relationship of age with perpetration and no relationship with victimization. A meta-analysis conducted by Barlett and Coyne (2014) that included 109 studies on cyberbullying showed that perpetration was slightly more common in boys than in girls but this difference was very small. They also found that girls were more involved in younger age groups and boys were more involved in older age groups. In a meta-analysis on cyberbullying with 131 empirical studies, Kowalski et al. (2014) found that there was a weak positive correlation of perpetration with age and no relationship in case of victimization. A systematic narrative review conducted by Tokunaga (2010) shows that most of the studies did not find gender differences in cyber-victimization rates and concludes that its prevalence with age could be curvilinear. According to the narrative review conducted by Farrington and Baldry (2010), direct perpetration is more common in boys and indirect perpetration seems to be more common among girls. The relationship of perpetration with age is not clear whereas victimization rates seem to drop in older children.

Findings on prevalence in relation to school location in rural or urban areas and different population sizes are also contradictory. O'Moore et al. (1997) found that in primary schools, there was more bullying in urban locations and in secondary education, prevalence was higher in rural zones. Wolke et al. (2001) found that there was more bullying in rural English schools. In a broad sample of more than 15.000 US students, Nansel et al. (2001) found that there was no difference in victimization in urban, sub-urban, town or rural locations and that there were slightly more students (3–5%) who reported perpetration in rural locations. Smokowski et al. (2013) found that the prevalence of victimization in rural areas was higher than the US national rate. Other studies report no differences between rural and urban locations (Olweus, 1993; Seals and Young, 2003). In Turkey, Akbulut et al. (2010) revealed that there was no significant difference among big cities, small cities, towns and villages in cyber-victimization rates.

Some studies focused also on bullying and cyberbullying in minority and majority groups, although research on the latter is still very scarce (Zych et al., 2015b). There are several meta-analyses on the topic that, again, report contradictory findings. Vitoroulis and Vaillancourt (2015) conducted a meta-analysis with 105 empirical studies and found no overall difference between ethnic majority and minorities. Nevertheless, when analyzed by country, minorities were more victimized in the UK whereas the majority groups were found to suffer more victimization in the US. A narrative systematic review of seven studies on minorities and cyberbullying (Hamm et al., 2015) found that the results are inconclusive, with some studies finding more involvement in minorities, others in majorities and others no differences between the groups.

Different empirical studies also found contradictory results. In adolescents from Spain and England, Monks et al. (2008) reported no difference in personal victimization and more cultural verbal victimization in the minorities. A study with Asian American adolescents shows that they are less bullied than other groups although the victimization rate is also higher for racist victimization (Cooc and Gee, 2014). There are also other studied that found no difference in victimization between majorities and minorities in general victimization but more discriminatory racist victimization in minorities (Durkin et al., 2012). On the other hand, a study with children including first and second generation immigrants in Finland (Strohmeier et al., 2011) shows that these groups suffered more victimization than the majority. In Spanish adolescents, Rodríguez-Hidalgo et al. (2014) found that the majority group suffered less peer victimization than the ethnic—cultural minority groups and that victimization of the minorities was even greater in case of racist victimization (e.g., racist insults). Wolke et al. (2001) found that, in English and German primary school pupils, there was no difference between the majority and ethnic minorities in perpetration but minorities were more victimized. In Flemish adolescents, Agirdag et al. (2011) found that for the native students, ethnic concentration in school did not predict victimization whereas for non-native adolescents, higher concentration of minorities predicted less peer-victimization. Thus, some studies show that ethnic-cultural minorities are more vulnerable to be involved in bullying and others show that there is no difference with the majority.

Results regarding sexual minorities are also inconclusive. Fedewa and Ahn (2011) meta-analyzed 18 studies on victimization in sexual minorities and found that sexually charged victimization was higher in this group when compared to the majority. Similarly, a meta-analysis conducted by Toomey and Russell (2016), also with 18 empirical studies, revealed that sexual minorities are more victimized than the heterosexual classmates. In a study conducted by Espelage et al. (2008) students who had doubts about their sexual orientation were more victimized then their heterosexual or LGB (lesbians, gays and bisexuals) peers. LGB youth reported more homophobic teasing than the sexual majority but there was no difference between the two in general victimization. In another study, when divided in subgroups and compared to the sexual majority, homosexual students of any gender reported more victimization, bisexual females reported more victimization and also more perpetration and gay males reported lower levels of perpetration (Berlan et al., 2010).

As described above, bullying and cyberbullying are serious problems present in schools all over the world. There are no specific profiles of children involved in the phenomena and findings regarding gender, age/grade or school location have been contradictory. Findings regarding the involvement of different minority and majority groups have also been contradictory and the number of studies on the topic is still scarce. Details related to the involvement of the minorities regarding gender, grade or school location are still needed. Thus, the current study has been conducted with the objective of describing the implication in bullying and cyberbullying victimization and perpetration of ethnic-cultural and sexual majority and minority students in Spain. The second objective was to find out whether this implication was different depending on gender, grade and school location (interaction). It is hypothesized that the minorities are more vulnerable than the majority group to be involved in bullying and cyberbullying. This was done with a representative sample of secondary education students in Andalusia.

## Methods

### Participants

The participants of this study were randomly selected from the population of 372,031 (2014/2015) secondary compulsory education students in Andalusia, Spain. Multi-stage stratified random sampling was used taking into account the proportion of student in each province (Almería, Cádiz, Córdoba, Granada, Huelva, Jaén, Málaga, and Sevilla), public and private schools and location in small (< 10,000 inhabitants), medium (10,000–100,000 inhabitants) and big cities/towns (>100,000 inhabitants). Schools were considered as clusters and it was estimated that selecting one line from each grade (1–4) in each school would give at least 80 students in each school (20 per classroom).

With these considerations, 22 schools were randomly selected to be included in this study. The total number of students was of 2139 which accounts for 95% of reliability and a sampling error of 2.1%. Among these students, 1088 were girls (50.9%), 1026 were boys (48.0%), their mean age was of 13.79 years (*SD* = 1.40) ranging from 11 to 19 (grade 1 *M* = 12.21, *SD* = 0.64; grade 2 *M* = 13.36, *SD* = 0.81; grade 3 *M* = 14.36, *SD* = 0.85; grade 4 *M* = 15.35, *SD* = 0.80). Students were equally distributed among the grades: 542 in the first grade (25.3%), 555 in the second grade (25.9%), 529 in the third grade (24.7%) and 508 in the fourth grade (23.7%).

Participants were classified into minority and majority groups. Taking into account their ethnic-cultural group, there were 1636 (76.5%) of students with Spanish nationality who did not identify themselves as Gypsies, first or second generation immigrants. Students who identified themselves as first generation immigrants were 136 (6.4%) and 178 (8.3%) indicated that one or both of their parents were immigrants and were classified as second generation immigrants. Among the minorities, 101 (4.7%) identified themselves as Gypsies and there were also 88 (4.2%) students who did not identify their ethnic-cultural group. Students were also asked to indicate their sexual orientation with 2021 (94.5%) students who identified themselves as heterosexual, 26 (1.2%) as homosexual, 22 (1.0%) as bisexual, 2 (0.1%) transsexual, 35 with doubts. These groups were classified as heterosexual majority 2021 (94.5%) and sexual minorities (all the others) 85 (4.8%).Thirty three students (1.5%) did not report their sexual orientation. A double minority group (24 students) included participants who identified themselves in an ethnic-cultural minority and also sexual minority (e.g., immigrant and homosexual).

### Design and procedure

This is an *ex post facto* transversal descriptive study conducted with a survey answered by a randomly selected representative sample of Andalusian adolescents. To increase representativeness regarding the time point in the academic year (the beginning, the end, the first or the second semester), data were collected in the second semester of the 2014/2015 and the first semester of the 2015/2016 academic years. After selecting the 22 schools, researchers contacted the head teachers providing information about the study and asking for their collaboration. After obtaining the permissions, researchers went to each school and explained the objectives of the study together with the instructions on the completion of the survey. Then, they asked students to fill in the questionnaires in about 30 min during their regular classroom hours. Participation was voluntary and totally anonymous and students were allowed to refuse to participate or withdraw in any moment (only 15 participants decided to do this). Teachers had no access to the questionnaires which were directly collected by the senior researchers responsible for the project. Procedure was approved by the ethic committee of the University of Cordoba.

### Instruments

First, students answered a series of questions on the sociodemographic variables such as gender, age, grade, ethnic-cultural group (an open question on the nationality of the student, country of origin of their mothers and fathers) and sexual orientation (checking a box next to heterosexual, homosexual, bisexual, transsexual or I have doubts). The meaning of each term was explained by the researchers before these questions were answered. Then, students were asked to fill in the following questionnaires:

- European Bullying Intervention Project Questionnaire (Brighi et al., 2012; Del Rey et al., 2012; Ortega-Ruiz et al., 2016) is a scale with 14 items divided into two factors: seven items for victimization and seven items for perpetration. These items are answered on a five point Likert scale ranging from 0 (never) to 4 (more than once a week) and in this study they referred to “the past few months.” This questionnaire shows very good Cronbach alphas in its validation study (Del Rey et al., 2012; victimization 0.84 and perpetration 0.73) and for the current sample (victimization α = 0.90, Ω = 0.90 and perpetration α = 0.90, Ω = 0.90). Also in this study, Confirmatory Factor Analysis shows very good adjustment of the data to this two factor structure (SB χ^2^ = 962.01; *df* = 76; NFI = 0.95; NNFI 0.94; CFI = 0.95; RMSEA = 0.076, 90% CI = 0.072–0.081).
- European Cyberbullying Intervention Project Questionnaire (Brighi et al., 2012; Del Rey et al., 2015; Ortega-Ruiz et al., 2016) is a questionnaire with 22 items also divided into two factors—11 for cyber-victimization and 11 for cyber-perpetration. These items are answered on a five point Likert scale ranging from 0 (never) to 4 (more than once a week) and in this study they referred to “the past few months.” This questionnaire shows excellent Cronbach alphas in its validation study (Ortega-Ruiz et al., 2016); victimization 0.80 and perpetration 0.88) and for the current sample (victimization α = 0.94, Ω = 0.94 and perpetration α = 0.96, Ω = 0.96). Also in the current study, Confirmatory Factor Analysis shows very good adjustment of the data to this two factor structure (SB χ^2^ = 1426.06; *df* = 208; NFI = 0.97; NNFI 0.97; CFI = 0.98; RMSEA = 0.054, 90% CI = 0.052–0.057).

### Data analysis

Reliability statistics (Cronbach's alphas and McDonald's omegas) for the questionnaires were calculated by means of the FACTOR software and Confirmatory Factor Analyses were performed with EQS 6.2 software to find out whether the factor structure is adequate for the data.

Students were classified to majority vs. minority groups. First, ethnic-cultural majority (Spanish nationality with no report of first or second generation immigrant or a Gypsy minority) was compared to the ethnic-cultural minority as a whole (first and second generation immigrants and Gypsy). Then, sexual majority (heterosexual) was compared to the sexual minority (LGBT and doubts). These groups were compared by means of the Student-*t*-test with SPSS 23 and effect sizes were calculated with Cohen's d on the Campbell Collaboration effect size calculator.

Later, specific groups such as majority (ethnic-cultural and sexual), sexual minority (LGBT and doubts), first generation immigrants, second generation immigrants, Gypsy and double minority (students who reported ethnic-cultural and sexual minority at the same time) were compared by means of ANOVA (Welch if variance was found to be heterogeneous). Pairwise Games-Howell comparisons were performed and effect sizes were calculated with Cohen's d. The latter is considered significant if the confidence intervals do not include 0. Although the *p* value is related to the effect size, it is affected by the number of participants in a group (Frías et al., 2000). Thus, in the current study, possible difference between the two (e.g., non-significant *p* and significant effect size) could be explained by the small or unequal number of participants in some groups. Specific subgroups (e.g., bisexual, homosexual, transsexual or immigrants from Latin America, Europe, Asia, etc.), were not compared because the number of participants in each group was considered too low to be statistically analyzed.

Hierarchical lineal regression analyses with main effects and interactions were performed. Interaction analyses were performed to find out whether minorities involvement in bullying or cyberbullying differ by gender, grade or location size (e.g., if minority boys are more or less victimized than minority girls or if minorities are more or less victimized in small or big cities). All these variables were dummy coded (0,1) where 0 was assigned as “no” and 1 as “yes” (e.g., majority − 0 = no, 1 = yes, boys − 0 = no, 1 = yes, etc.). To avoid the dummy variable trap, redundant variables such as girls, big location size and grade 1 were not included. Involvement in bullying and cyberbullying was treated as continuous variables and therefore, it refers to the total score on victimization and perpetration but without classifying participants as victims or bullies.

## Results

First all the ethnic-cultural minorities grouped in one (*n* = 413) were compared to the ethnic-cultural majority group (*n* = 1630) in their involvement in bullying and cyberbullying perpetration and victimization. The results show no difference in bullying victimization [minority *M* = 4.74; *SD* = 5.75 and majority *M* = 4.19; *SD* = 5.08; *t*_(586.09)_ = 1.79, *p* = 0.07] or cyber-victimization [minority *M* = 3.37; *SD* = 5.24 and majority *M* = 2.84; *SD* = 4.67; *t*_(572.34)_ = 1.87, *p* = 0.06]. Minorities were found to be more involved in bullying perpetration [minority *M* = 2.83; *SD* = 4 and majority *M* = 2.26; *SD* = 3.54; *t*_(585.12)_ = 2.64, *p* < 0.01; *d* = 0.16, 95% CI = 0.05–0.26] but there was no significant difference in cyber-perpetration [minority *M* = 1.91; *SD* = 3.79 and majority *M* = 1.65; *SD* = 3.81; *t*_(2014)_ = 1.24, *p* = 0.22].

Second, all sexual minorities grouped in one (*n* = 83) were compared to the sexual majority (*n* = 2015) in relation to bullying and cyberbullying perpetration and victimization. It was found that bullying victimization [minority *M* = 6.90; *SD* = 6.46 and majority *M* = 4.23; *SD* = 5.23; *t*_(86.48)_ = 3.72, *p* < 0.01; *d* = 0.51, 95% CI = 0.29–0.73] and cyber-victimization [minority *M* = 5.29; *SD* = 7.53 and majority *M* = 2.91; *SD* = 4.80; *t*_(84.81)_ = 2.86, *p* < 0.01; *d* = 0.48, 95% CI = 0.26–0.70] were both higher in sexual minorities. In bullying perpetration, there was no difference between sexual minorities and majority [minority *M* = 3.12; *SD* = 4.78 and majority *M* = 2.37; *SD* = 3.67; *t*_(86.02)_ = 1.41, *p* = 0.16] and sexual minorities scored higher in cyber-perpetration [minority *M* = 3.30; *SD* = 6.55 and majority *M* = 1.65; *SD* = 3.76; *t*_(84.27)_ = 2.29, *p* < 0.05; *d* = 0.42, 95% CI = 0.20–0.64].

After comparing the minorities grouped altogether in big groups, we conducted also analysis to find out which particular minorities differed in their implication in bullying and cyberbullying. Students were classified as majority when they were not from ethnically-cultural minority group (not first or second immigrants or Gypsies) and were not from the sexual minority group. Double minority students were those who were from one of the ethnic-cultural group and also sexual minority group. The results are presented in Table 1.

**Table 1:** **Implication in bullying and cyberbullying roles of the majority group, first and second generation immigrants, Gypsies and sexual minorities**.

	**Majority *n* = 1555 *M* (*SD*)**	**1st generation immigrants *n* = 126 *M* (*SD*)**	**2nd generation immigrants *n* = 167 *M* (*SD*)**	**Gypsies *n* = 96 *M* (*SD*)**	**Sexual minority *n* = 59 *M* (*SD*)**	**Double minority *n* = 24 *M* (*SD*)**	**Total *n* = 2027 *M* (*SD*)**	***F*_(5, 143)_**
Bullying victimization	4.11 (5.04)	4.93 (6.29)	4.55 (5.50)	4.04 (4.77)	6.51 (6.21)	7.88 (7.09)	4.31 (5.25)	3.43^**^
Bullying perpetration	2.25 (3.52)	2.37 (3.52)	2.65 (3.64)	3.40 (4.44)	2.71 (4.07)	4.13 (6.19)	2.38 (3.64)	2.02
Cyberbullying victimization	2.78 (4.59)	3.05 (4.49)	2.87 (4.91)	4.03 (5.96)	5.05 (7.73)	5.88 (7.15)	2.96 (4.87)	2.62^*^
Cyberbullying perpetration	1.60 (3.68)	2.02 (3.99)	1.55 (2.96)	1.94 (3.91)	3.12 (6.74)	3.75 (6.17)	1.71 (3.83)	2.48

* *p < 0.05*,

** p < 0.01.

As shown in Table 1, there are no significant differences among the groups in bullying or cyberbullying perpetration. On the other hand, there are significant differences in both, bullying and cyberbullying victimization. Taking into account that the Levene test show unequal variances in both variables (*p* < 0.01), Games-Howell pairwise *post-hoc* comparisons were performed to find out which groups differed in victimization. Through Games-Howell comparisons, significant differences were found only between majority and sexual minority in bullying victimization (*M* = 4.11, *SD* = 5.04 vs. 6.51; *SD* = 6.21; *p* = 0.05).

Taking into account unequal and sometimes small number of participants in the groups, also Cohen's d with confidence intervals were calculated to find other possible differences among the groups. For bullying victimization, significant differences were found between majority and sexual minority (*d* = 0.47; 95% CI = 0.21–0.73), majority and double minority (*d* = 0.74; 95% CI = 0.34–1.15). For bullying perpetration, differences were found between majority and Gypsies (*d* = 0.32; 95% CI = 0.11–0.53) and also majority and double minority (*d* = 0.53; 95% CI = 0.12–0.93). For cyberbullying victimization, significant differences were found between majority and Gypsies (*d* = 0.27; 95% CI = 0.06–0.47), majority and sexual minorities (*d* = 0.48; 95% CI = 0.22–0.74) and majority with double minority (*d* = 0.67; 95% CI = 0.26–1.07). For cyberbullying perpetration, significant differences were found between the majority and sexual minorities (*d* = 0.40; 95% CI = 0.14–0.66) and the majority with double minority (*d* = 0.58; 95% CI = 0.17–0.98).

To find out if minority or majority groups and their interaction with gender, setting (small, medium or big towns/cities) and grade predicted involvement in bullying and cyberbullying victimization and perpetration, hierarchical linear regression analyses were performed. Variables were dummy coded (0, 1) and then, location size, grade and gender were entered in Block 1, minority groups in Block 2 and interactions in Block 3 (see Table 2).

**Table 2:** **Hierarchical lineal regression analysis predicting bullying and cyberbullying victimization and perpetration taking into account majority and minority groups, gender, location size and grade**.

	**Bullying**				**Cyberbullying**			
	**Victimization**		**Perpetration**		**Victimization**		**Perpetration**	
	**Δ*R*^2^**	**β**	**Δ*R*^2^**	**β**	**Δ*R*^2^**	**β**	**Δ*R*^2^**	**β**
MODEL 1	0.01^**^		0.05^**^		0.01^**^		0.02^**^	
Middle size		−0.06^**^		−0.06^*^		−0.10^**^		−0.06^**^
Small size		0.02		−0.04		−0.01		−0.02
Grade 2		0.00		0.03		0.03		0.03
Grade 3		−0.05		0.03		0.02		0.05
Grade 4		−0.08^**^		0.08^**^		0.04		0.10^**^
Boys		0.01		0.19^**^		0.02		0.09^**^
MODEL 2	0.01^**^		0.01^*^		0.01^**^		0.01^**^	
Immi1st		0.04		−0.01		0.01		0.02
Immi2nd		0.02		0.02		0.01		−0.01
Gypsy		−0.01		0.05^*^		0.05^*^		0.02
Sexual minority		0.08^**^		0.02		0.07^**^		0.07^**^
Double minority		0.08^**^		0.05^*^		0.07^**^		0.06^**^
MODEL 3	0.02		0.03^**^		0.02		0.03^**^	
Middle size		0.03		0.11		0.21^*^		0.18
Small size		0.20		−0.07		0.04		0.03
Grade 2		0.06		−0.13		−0.07		−0.05
Grade 3		−0.02		−0.06		−0.01		0.11
Grade 4		−0.04		−0.06		0.06		−0.03
Boys		0.03		0.33^**^		−0.05		−0.01
Immi1st		0.14^*^		−0.00		−0.01		0.00
Immi2nd		0.04		0.07		0.05		0.04
Gypsy		−0.01		−0.01		0.08		0.00
Sexual minority		0.13^*^		−0.00		−0.10		−0.16^**^
Double minority		−0.08		−0.21^**^		−0.08		−0.15^**^
Majority^*^boys		−0.02		−0.13		0.08		0.10
Immi1st^*^boys		0.01		−0.06		0.01		0.04
Immi2nd^*^boys		−0.01		−0.12^**^		−0.02		−0.03
Gypsy^*^boys		0.01		−0.02		−0.00		0.04
Sexual min^*^boys		−0.03		−0.05		0.04		0.07
Double^*^boys		0.02		0.09^*^		0.07		0.15^**^
Majority^*^middle		−0.09		−0.18		−0.30^**^		−0.24^*^
Majority^*^small		−0.16		0.03		−0.04		−0.05
Immi1st^*^middle		−0.07		−0.09^*^		−0.12^**^		−0.11^**^
Immi1st^*^small		−0.03		0.03		−0.01		−0.02
Immi2nd^*^middle		−0.01		−0.04		−0.09^*^		−0.09^*^
Immi2nd^*^small		−0.04		0.03		−0.03		−0.00
Gypsy^*^middle		0.02		0.01		−0.06		−0.04
Gypsy^*^small		−0.08^*^		−0.01		−0.01		−0.02
Sex min^*^middle		−0.05		0.01		−0.00		−0.06
Sex min^*^small		−0.02		0.04		0.02		0.03
Double^*^middle		0.01		0.07		−0.01		0.03
Double^*^small		−0.07^*^		−0.07^*^		−0.05		−0.06
Majority^*^Grade2		−0.05		0.16		0.10		0.07
Majority^*^Grade3		−0.02		0.09		0.02		−0.07
Majority^*^Grade4		−0.03		0.12		−0.04		0.09
Immi1st^*^Grade2		−0.08		0.05		0.04		0.03
Immi1st^*^Grade3		−0.03		0.04		0.06		−0.01
Immi1st^*^Grade4		−0.08		0.07		0.03		0.08
Immi2nd^*^Grade2		−0.01		0.07		0.02		0.04
Immi2nd^*^Grade3		−0.03		−0.01		0.00		−0.02
Immi2nd^*^Grade4		−0.05		0.01		−0.04		0.03
Gypsy^*^Grade2		−0.02		0.05		0.01		0.01
Gypsy^*^Grade3		−0.02		0.04		−0.03		−0.04
Gypsy^*^Grade4		−0.05		0.05		0.01		0.07
Sex min^*^Grade2		−0.04		0.00		0.07		0.09^*^
Sex min^*^Grade3		−0.01		0.03		0.09^*^		0.06
Sex min^*^Grade4		−0.02		0.03		0.09		0.10^*^
Double^*^Grade2		0.12^*^		0.13^**^		0.08^*^		0.07
Double^*^Grade3		0.08^*^		0.09^**^		0.08^*^		0.05
Double^*^Grade4		0.08^*^		0.14^**^		0.06		0.11^**^
TOTAL	0.05^**^		0.08^**^		0.04^**^		0.06^**^	

* *p < 0.05*,

** p < 0.01; adjusted R^2^ for bullying victimization = 0.02; bullying perpetration = 0.06; cyberbullying victimization = 0.02, and cyberbullying perpetration = 0.04. Small size, locations with less than 10,000 inhabitants; Middle size, locations with 10,000–100,000 inhabitants; Immi1st, first generation immigrants; Immi 2nd, second generation immigrants; Sexual min, sexual minorities; Double, sexual and ethnic-cultural minority.

The regression analysis shows that the amount of variance in bullying and cyberbullying perpetration and victimization predicted by the location size, grade and gender is low (1%, 5%, 1% and 2%, respectively) but significant. Lower level of bullying and cyberbullying victimization and perpetration is predicted by middle size location (β = −0.06, *p* < 0.01, β = −0.06, *p* < 0.05, β = −0.10, *p* < 0.01 and β = −0.06, *p* < 0.01; respectively). Being in grade 4 predicts lower bullying victimization (β = −0.08, *p* < 0.01) but higher bullying perpetration (β = 0.08, *p* < 0.01) and higher cyberbullying perpetration (β = 0.10, *p* < 0.01). Being a boy predicts higher level of perpetration in bullying (β = 0.19, *p* < 0.01) and cyberbullying (β = 0.09, *p* < 0.01).

Being in a minority group predicts bullying and cyberbullying victimization and perpetration above and beyond the demographic variables included in the Block 1. Nevertheless, the increase in the amount of variance, although significant, is low (1% in each dependent variable). Being in the Gypsy group predicts higher level of bullying perpetration (β = 0.05, *p* < 0.05) and cyberbullying victimization (β = 0.05, *p* < 0.05). Being in a sexual minority predicts higher level of bullying victimization (β = 0.08, *p* < 0.01), cyberbullying victimization (β = 0.07, *p* < 0.01), and cyberbullying perpetration (β = 0.07, *p* < 0.01). Being in a double minority (ethnic-cultural and sexual at the same time) predicts higher level of bullying and cyberbullying victimization and perpetration (β = 0.08, *p* < 0.01, β = 0.05, *p* < 0.05, β = 0.07, *p* < 0.01 and β = 0.06, *p* < 0.01; respectively).

Adding interactions in the Block 3 predicts bullying and cyberbullying perpetration (but not victimization) above and beyond the demographic variables and being in a minority group. Again, the increase in the amount of variance is significant but low (3% in both cases). Being a second generation immigrant and boy predicts lower level of bullying perpetration (β = −0.12, *p* < 0.01). Being a boy in a double minority predicts higher level of perpetration in bullying (β = 0.09, *p* < 0.05) and cyberbullying (β = 0.15, *p* < 0.01). Being in the majority group or second generation immigrant in a middle size location predicts lower levels of cyberbullying victimization (β = −0.30, *p* < 0.01, β = − 0.09, *p* < 0.01; respectively) and perpetration (β = −0.24, *p* < 0.05, β = −0.09, *p* < 0.01; respectively). Being a first generation immigrant in middle size location predicts lower levels of bullying perpetration (β = −0.09, *p* < 0.05) and cyberbullying victimization (β = −0.12, *p* < 0.01) and perpetration (β = −0.11, *p* < 0.01). Being in the Gypsy group in a small location predicts lower level of bullying victimization (β = −0.08, *p* < 0.01). Being in a double minority in a small location predicts lower bullying victimization (β = −0.07, *p* < 0.05) and perpetration (β = −0.07, *p* < 0.05). Sexual minority in grade 2 and 4 predicts more cyberbullying perpetration (β = 0.09, *p* < 0.05, β = 0.10, *p* < 0.05; respectively), in grade 3 predicts more cyberbullying victimization (β = 0.09, *p* < 0.05). Double minority in grade 2 and 3 predicts higher levels of bullying victimization (β = 0.12, *p* < 0.01, β = 0.08, *p* < 0.05; respectively) and perpetration (β = 0.13, *p* < 0.01, β = 0.09, *p* < 0.01; respectively) and cyberbullying victimization (β = 0.08, *p* < 0.01, β = 0.08, *p* < 0.01; respectively). Grade 4 and double minority predicts higher bullying victimization (β = 0.08, *p* < 0.05) and perpetration (β = 0.14, *p* < 0.01) and cyberbullying perpetration (β = 0.11, *p* < 0.01).

## Discussion

Bullying and cyberbullying are extremely damaging types of interpersonal violence present in schools throughout different countries and regions (Zych et al., 2015a). Implication in these phenomena leads to very serious consequences such as violence (Ttofi et al., 2012), offending (Ttofi et al., 2011a) or drug use (Ttofi et al., 2016) later in life (Ttofi et al., 2011b). Given the fact that these problems are still present and prevalent (Zych et al., 2016), it is important to advance knowledge on the topic to eradicate bullying and cyberbullying.

Cultural, ethnic or sexual diversity is present in schools (Llorent-Bedmar, 2013) and the societies are concerned with inclusive education and not leaving any child behind due to the inadequate response to their needs (Ainscow et al., 2006). Thus, it is crucial to conduct research specifically focused on these possibly vulnerable minority groups. Studies on bullying and cyberbullying in this context are still very scarce (see the review conducted by Zych et al., 2015b). Thus, the objectives of this study were to describe the involvement in bullying and cyberbullying victimization and perpetration of students from the minority and majority groups and find out whether the involvement can be predicted by the group in relation to gender, grade and school location. This study was done with a representative sample of adolescents from southern Spain (Andalusia).

When all the ethnic-cultural minorities were treated as one group, it was found that there was no difference in bullying and cyberbullying victimization in comparison to the majority group. These findings are similar to those reported in other studies (Monks et al., 2008; Durkin et al., 2012; Vitoroulis and Vaillancourt, 2015). There was also no difference in cyberbullying perpetration but the minority was found to be more involved in bullying perpetration. For sexual minorities, results show the opposite pattern. In comparison to the majority, they were found to be more victimized (bullying and cyberbullying) and there was no difference in bullying perpetration. On the other hand, sexual minorities were found to be more involved in cyberbullying perpetration. Very few studies were conducted on this topic but previous research found that sexually charged victimization was more frequent in this group (Fedewa and Ahn, 2011), findings that are in line with the current results.

When groups were separated in different minorities, sexual minority was found to be the most vulnerable to be victimized through bullying. When differences were calculated through Cohen's d, it was found that sexual and double minorities were more victimized through bullying and cyberbullying than the majority. In case of cyberbullying, Gypsies were also found to be more victimized. Double minority and Gypsies were also found to be more involved in bullying perpetration and both, sexual and double minorities were more involved in cyberbullying perpetration. Thus, the current study shows that some minorities (especially sexual minorities) are indeed more vulnerable to be involved in bullying and cyberbullying. These results are similar to some previously reported findings (Wolke et al., 2001; Strohmeier et al., 2011; Rodríguez-Hidalgo et al., 2014).

Prediction analyses show that being in the majority or minority group predicts a small (but significant) amount of variance of the involved in bullying or cyberbullying victimization and perpetration. Being in the Gypsy group predicts more bullying perpetration and cyberbullying victimization; sexual minority predicts more bullying and cyberbullying victimization and cyberbullying perpetration and double minority predicts more bullying and cyberbullying victimization and perpetration. Being a boy in the double minority predicts more perpetration, findings in line with other research that points out that the frequency of perpetration is higher in boys (Cook et al., 2010; Barlett and Coyne, 2014). Location in the middle size towns predicts less implication in cyberbullying of the majority group and first and second generation immigrants. It also predicts less bullying perpetration in first generation immigrants. Location in small towns predicts less bullying victimization in Gypsies and less bullying victimization and perpetration in double minorities. Results show also interaction with being enrolled in different grades (1–4). In grades 2 and 4, sexual minorities are involved in more cyberbullying perpetration, and in grade 3 in more cyber-victimization. Double minorities more involved in bullying victimization and perpetration in grades 2, 3, and 4 whereas in cyberbullying, they are more involved in victimization in grades 2 and 3 and in perpetration in grade 4.

All the patterns and interactions found in this study should be taken into account when identifying the most vulnerable minority groups. At the same time, they should be interpreted with caution given the small amount of explained variance. Other limitations are related to the fact that the socio-economic status of the families, parental styles, social and emotional competencies or access to the information and communication technologies were not controlled for. These variables were found to be important in different studies on bullying and cyberbullying (Gómez-Ortiz et al., 2014, 2016; Herrera López et al., 2016; Romera et al., 2016). Specific minority sub-groups could not be compared (e.g., immigrants from different countries or different sexual minorities) due to the low number of participants in each group. All these questions could be answered in future studies. Future research should also focus on whole-school policy, inclusive education and school management strategies that could eradicate bullying in all the ethnic-cultural groups. It could be interesting to discover, for example, which strategies and policies are the most effective in culturally diverse settings.

The findings of this study show that minorities are more vulnerable than the majority to be involved in bullying and cyberbullying. These findings should have implications for educational policy and practice. It is important to promote inclusion, *convivencia* and *cyberconvivencia* among all the minority and majority groups so that no child is left behind. Previous studies found that increase in ethnic concentration did not affect the majority but was related to less victimization in the minorities (Agirdag et al., 2011). Thus, it is possible that more diverse and inclusive school settings, in which all the groups are respected and cared for, in which educators and policy makers are able to respond to the needs of each and every student would make it possible to eradicate bullying and cyberbullying.

## Author contributions

All authors made substantial contribution to the theoretical framework, design, data collection or interpretation of this study. All contributed to this article and approved its publication.

### Conflict of interest statement

The authors declare that the research was conducted in the absence of any commercial or financial relationships that could be construed as a potential conflict of interest. The reviewers AD and DA and handling Editor declared their shared affiliation, and the handling Editor states that the process nevertheless met the standards of a fair and objective review.

## Footnotes

^1^Convivencia, in Spanish (valid for the Kingdom of Spain and all the Spanish-speaking countries) is a term referring to the positive school climate including interpersonal relationships of the students and teachers with each other, their peers, family and community; based on the principles of equality and respect. Ciberconvivencia is based on the same educational principles but in the computer-mediated communication.
